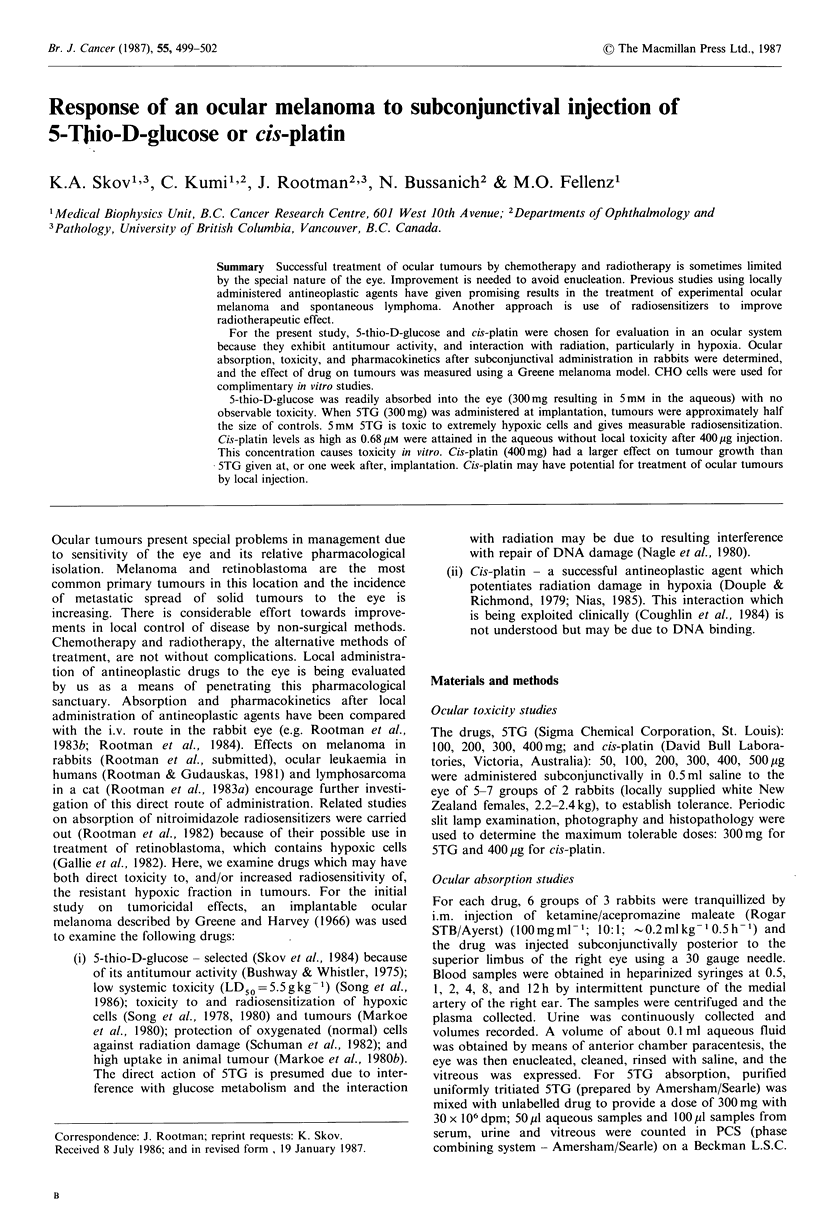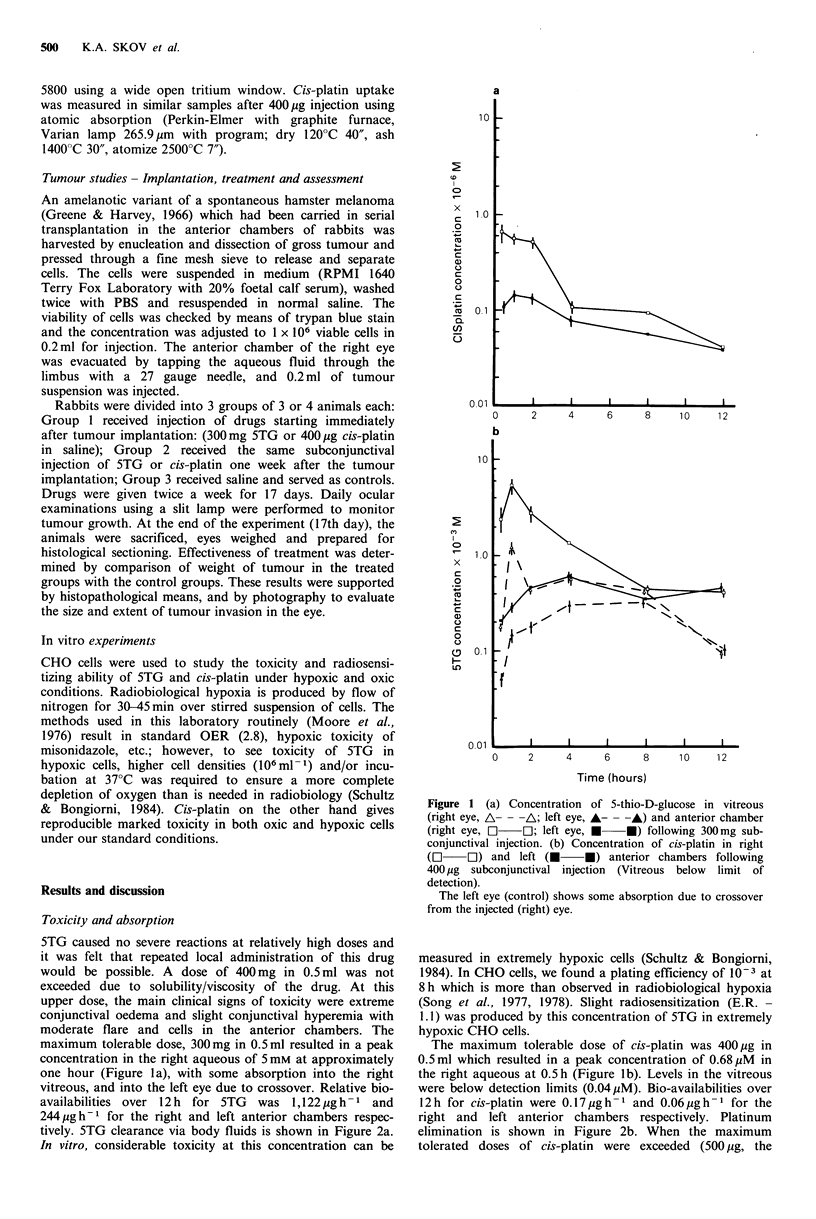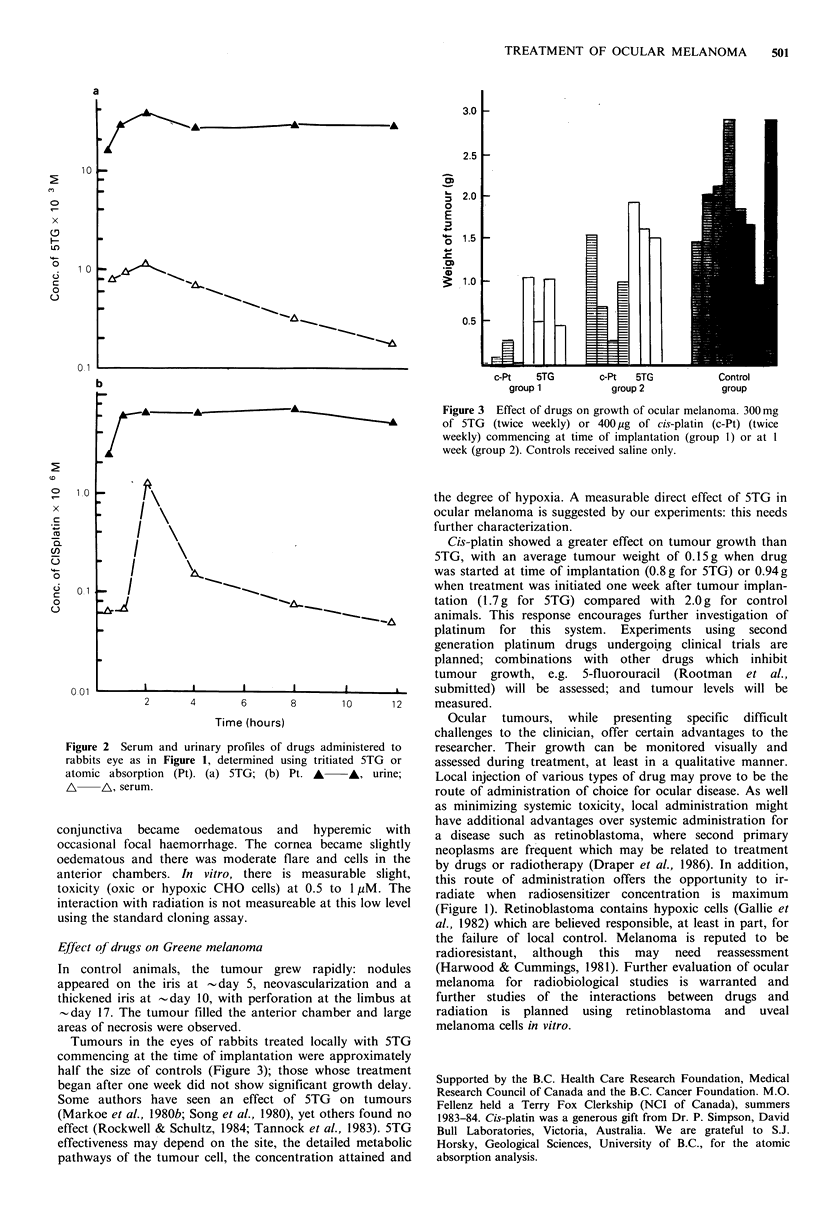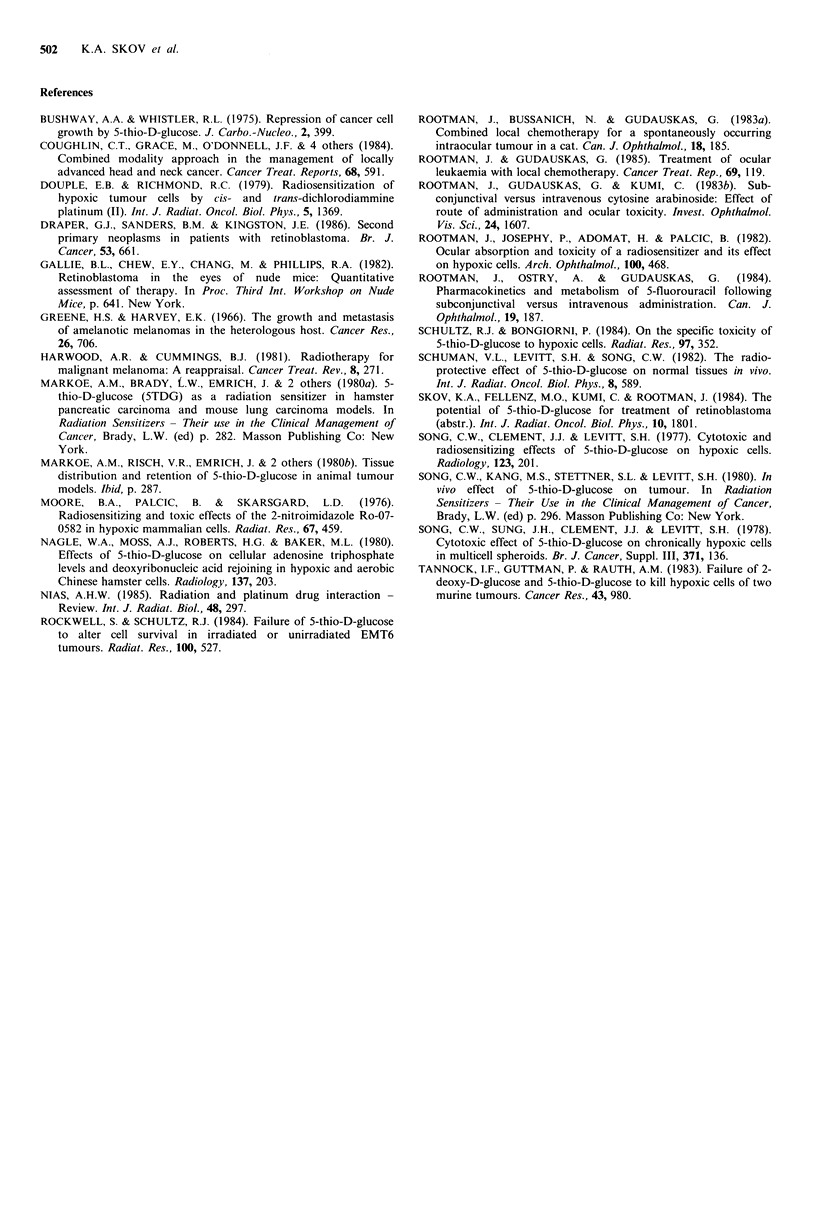# Response of an ocular melanoma to subconjunctival injection of 5-thio-D-glucose or cis-platin.

**DOI:** 10.1038/bjc.1987.101

**Published:** 1987-05

**Authors:** K. A. Skov, C. Kumi, J. Rootman, N. Bussanich, M. O. Fellenz

## Abstract

Successful treatment of ocular tumours by chemotherapy and radiotherapy is sometimes limited by the special nature of the eye. Improvement is needed to avoid enucleation. Previous studies using locally administered antineoplastic agents have given promising results in the treatment of experimental ocular melanoma and spontaneous lymphoma. Another approach is use of radiosensitizers to improve radiotherapeutic effect. For the present study, 5-thio-D-glucose and cis-platin were chosen for evaluation in an ocular system because they exhibit antitumour activity, and interaction with radiation, particularly in hypoxia. Ocular absorption, toxicity, and pharmacokinetics after subconjunctival administration in rabbits were determined, and the effect of drug on tumours was measured using a Greene melanoma model. CHO cells were used for complimentary in vitro studies. 5-thio-D-glucose was readily absorbed into the eye (300 mg resulting in 5 mM in the aqueous) with no observable toxicity. When 5TG (300 mg) was administered at implantation, tumours were approximately half the size of controls. 5 mM 5TG is toxic to extremely hypoxic cells and gives measurable radiosensitization. Cis-platin levels as high as 0.68 microM were attained in the aqueous without local toxicity after 400 micrograms injection. This concentration causes toxicity in vitro. Cis-platin (400 mg) had a larger effect on tumour growth than 5TG given at, or one week after, implantation. Cis-platin may have potential for treatment of ocular tumours by local injection.


*Br. J. Cancer* (1987), **55**, 499–502 © The Macmillan Press Ltd., 1987

### Response of an ocular melanoma to subconjunctival injection of 5-Thio-D-glucose or *cis*-platin

K.A. Skov[1,3], C. Kumi[1,2], J. Rootman[2,3], N. Bussanich[2] & M.O. Fellenz[1]

[1]*Medical Biophysics Unit, B.C. Cancer Research Centre, 601 West 10th Avenue;* [2]*Departments of Ophthalmology and* [3]*Pathology, University of British Columbia, Vancouver, B.C. Canada.*

**Summary** Successful treatment of ocular tumours by chemotherapy and radiotherapy is sometimes limited by the special nature of the eye. Improvement is needed to avoid enucleation. Previous studies using locally administered antineoplastic agents have given promising results in the treatment of experimental ocular melanoma and spontaneous lymphoma. Another approach is use of radiosensitizers to improve radiotherapeutic effect.

For the present study, 5-thio-D-glucose and *cis*-platin were chosen for evaluation in an ocular system because they exhibit antitumour activity, and interaction with radiation, particularly in hypoxia. Ocular absorption, toxicity, and pharmacokinetics after subconjunctival administration in rabbits were determined, and the effect of drug on tumours was measured using a Greene melanoma model. CHO cells were used for complimentary *in vitro* studies.

5-thio-D-glucose was readily absorbed into the eye (300 mg resulting in 5 mM in the aqueous) with no observable toxicity. When 5TG (300 mg) was administered at implantation, tumours were approximately half the size of controls. 5 mM 5TG is toxic to extremely hypoxic cells and gives measurable radiosensitization. *Cis*-platin levels as high as 0.68 $\mu$M were attained in the aqueous without local toxicity after 400 $\mu$g injection. This concentration causes toxicity *in vitro*. *Cis*-platin (400 mg) had a larger effect on tumour growth than 5TG given at, or one week after, implantation. *Cis*-platin may have potential for treatment of ocular tumours by local injection.

Ocular tumours present special problems in management due to sensitivity of the eye and its relative pharmacological isolation. Melanoma and retinoblastoma are the most common primary tumours in this location and the incidence of metastatic spread of solid tumours to the eye is increasing. There is considerable effort towards improvements in local control of disease by non-surgical methods. Chemotherapy and radiotherapy, the alternative methods of treatment, are not without complications. Local administration of antineoplastic drugs to the eye is being evaluated by us as a means of penetrating this pharmacological sanctuary. Absorption and pharmacokinetics after local administration of antineoplastic agents have been compared with the i.v. route in the rabbit eye (e.g. Rootman *et al.*, 1983*b*; Rootman *et al.*, 1984). Effects on melanoma in rabbits (Rootman *et al.*, submitted), ocular leukaemia in humans (Rootman & Gudauskas, 1981) and lymphosarcoma in a cat (Rootman *et al.*, 1983*a*) encourage further investigation of this direct route of administration. Related studies on absorption of nitroimidazole radiosensitizers were carried out (Rootman *et al.*, 1982) because of their possible use in treatment of retinoblastoma, which contains hypoxic cells (Gallie *et al.*, 1982). Here, we examine drugs which may have both direct toxicity to, and/or increased radiosensitivity of, the resistant hypoxic fraction in tumours. For the initial study on tumoricidal effects, an implantable ocular melanoma described by Greene and Harvey (1966) was used to examine the following drugs:

(i) 5-thio-D-glucose – selected (Skov *et al.*, 1984) because of its antitumour activity (Bushway & Whistler, 1975); low systemic toxicity ($LD_{50} = 5.5\,g\,kg^{-1}$) (Song *et al.*, 1986); toxicity to and radiosensitization of hypoxic cells (Song *et al.*, 1978, 1980) and tumours (Markoe *et al.*, 1980); protection of oxygenated (normal) cells against radiation damage (Schuman *et al.*, 1982); and high uptake in animal tumour (Markoe *et al.*, 1980*b*). The direct action of 5TG is presumed due to interference with glucose metabolism and the interaction with radiation may be due to resulting interference with repair of DNA damage (Nagle *et al.*, 1980).

(ii) *Cis*-platin – a successful antineoplastic agent which potentiates radiation damage in hypoxia (Douple & Richmond, 1979; Nias, 1985). This interaction which is being exploited clinically (Coughlin *et al.*, 1984) is not understood but may be due to DNA binding.

### Materials and methods

### *Ocular toxicity studies*

The drugs, 5TG (Sigma Chemical Corporation, St. Louis): 100, 200, 300, 400 mg; and *cis*-platin (David Bull Laboratories, Victoria, Australia): 50, 100, 200, 300, 400, 500 $\mu$g were administered subconjunctivally in 0.5 ml saline to the eye of 5–7 groups of 2 rabbits (locally supplied white New Zealand females, 2.2–2.4 kg), to establish tolerance. Periodic slit lamp examination, photography and histopathology were used to determine the maximum tolerable doses: 300 mg for 5TG and 400 $\mu$g for *cis*-platin.

### *Ocular absorption studies*

For each drug, 6 groups of 3 rabbits were tranquillized by i.m. injection of ketamine/acepromazine maleate (Rogar STB/Ayerst) ($100\,mg\,ml^{-1}$; 10:1; $\sim 0.2\,ml\,kg^{-1}\,0.5\,h^{-1}$) and the drug was injected subconjunctivally posterior to the superior limbus of the right eye using a 30 gauge needle. Blood samples were obtained in heparinized syringes at 0.5, 1, 2, 4, 8, and 12 h by intermittent puncture of the medial artery of the right ear. The samples were centrifuged and the plasma collected. Urine was continuously collected and volumes recorded. A volume of about 0.1 ml aqueous fluid was obtained by means of anterior chamber paracentesis, the eye was then enucleated, cleaned, rinsed with saline, and the vitreous was expressed. For 5TG absorption, purified uniformly tritiated 5TG (prepared by Amersham/Searle) was mixed with unlabelled drug to provide a dose of 300 mg with $30 \times 10^6$ dpm; 50 $\mu$l aqueous samples and 100 $\mu$l samples from serum, urine and vitreous were counted in PCS (phase combining system – Amersham/Searle) on a Beckman L.S.C.

Correspondence: J. Rootman; reprint requests: K. Skov.
Received 8 July 1986; and in revised form , 19 January 1987.

5800 using a wide open tritium window. *Cis*-platin uptake was measured in similar samples after 400 $\mu$g injection using atomic absorption (Perkin-Elmer with graphite furnace, Varian lamp 265.9 $\mu$m with program; dry 120°C 40″, ash 1400°C 30″, atomize 2500°C 7″).

*Tumour studies – Implantation, treatment and assessment*

An amelanotic variant of a spontaneous hamster melanoma (Greene & Harvey, 1966) which had been carried in serial transplantation in the anterior chambers of rabbits was harvested by enucleation and dissection of gross tumour and pressed through a fine mesh sieve to release and separate cells. The cells were suspended in medium (RPMI 1640 Terry Fox Laboratory with 20% foetal calf serum), washed twice with PBS and resuspended in normal saline. The viability of cells was checked by means of trypan blue stain and the concentration was adjusted to $1 \times 10^6$ viable cells in 0.2 ml for injection. The anterior chamber of the right eye was evacuated by tapping the aqueous fluid through the limbus with a 27 gauge needle, and 0.2 ml of tumour suspension was injected.

Rabbits were divided into 3 groups of 3 or 4 animals each: Group 1 received injection of drugs starting immediately after tumour implantation: (300 mg 5TG or 400 $\mu$g *cis*-platin in saline); Group 2 received the same subconjunctival injection of 5TG or *cis*-platin one week after the tumour implantation; Group 3 received saline and served as controls. Drugs were given twice a week for 17 days. Daily ocular examinations using a slit lamp were performed to monitor tumour growth. At the end of the experiment (17th day), the animals were sacrificed, eyes weighed and prepared for histological sectioning. Effectiveness of treatment was determined by comparison of weight of tumour in the treated groups with the control groups. These results were supported by histopathological means, and by photography to evaluate the size and extent of tumour invasion in the eye.

In vitro *experiments*

CHO cells were used to study the toxicity and radiosensitizing ability of 5TG and *cis*-platin under hypoxic and oxic conditions. Radiobiological hypoxia is produced by flow of nitrogen for 30–45 min over stirred suspension of cells. The methods used in this laboratory routinely (Moore *et al.*, 1976) result in standard OER (2.8), hypoxic toxicity of misonidazole, etc.; however, to see toxicity of 5TG in hypoxic cells, higher cell densities ($10^6$ ml$^{-1}$) and/or incubation at 37°C was required to ensure a more complete depletion of oxygen than is needed in radiobiology (Schultz & Bongiorni, 1984). *Cis*-platin on the other hand gives reproducible marked toxicity in both oxic and hypoxic cells under our standard conditions.

**Figure 1** (a) Concentration of 5-thio-D-glucose in vitreous (right eye, △– – –△; left eye, ▲– – –▲) and anterior chamber (right eye, □——□; left eye, ■——■) following 300 mg subconjunctival injection. (b) Concentration of *cis*-platin in right (□——□) and left (■——■) anterior chambers following 400 $\mu$g subconjunctival injection (Vitreous below limit of detection).

The left eye (control) shows some absorption due to crossover from the injected (right) eye.

### Results and discussion

*Toxicity and absorption*

5TG caused no severe reactions at relatively high doses and it was felt that repeated local administration of this drug would be possible. A dose of 400 mg in 0.5 ml was not exceeded due to solubility/viscosity of the drug. At this upper dose, the main clinical signs of toxicity were extreme conjunctival oedema and slight conjunctival hyperemia with moderate flare and cells in the anterior chambers. The maximum tolerable dose, 300 mg in 0.5 ml resulted in a peak concentration in the right aqueous of 5 mM at approximately one hour (Figure 1a), with some absorption into the right vitreous, and into the left eye due to crossover. Relative bioavailabilities over 12 h for 5TG was 1,122 $\mu$g h$^{-1}$ and 244 $\mu$g h$^{-1}$ for the right and left anterior chambers respectively. 5TG clearance via body fluids is shown in Figure 2a. *In vitro*, considerable toxicity at this concentration can be measured in extremely hypoxic cells (Schultz & Bongiorni, 1984). In CHO cells, we found a plating efficiency of $10^{-3}$ at 8 h which is more than observed in radiobiological hypoxia (Song *et al.*, 1977, 1978). Slight radiosensitization (E.R. – 1.1) was produced by this concentration of 5TG in extremely hypoxic CHO cells.

The maximum tolerable dose of *cis*-platin was 400 $\mu$g in 0.5 ml which resulted in a peak concentration of 0.68 $\mu$M in the right aqueous at 0.5 h (Figure 1b). Levels in the vitreous were below detection limits (0.04 $\mu$M). Bio-availabilities over 12 h for *cis*-platin were 0.17 $\mu$g h$^{-1}$ and 0.06 $\mu$g h$^{-1}$ for the right and left anterior chambers respectively. Platinum elimination is shown in Figure 2b. When the maximum tolerated doses of *cis*-platin were exceeded (500 $\mu$g, the

**Figure 2** Serum and urinary profiles of drugs administered to rabbits eye as in **Figure 1**, determined using tritiated 5TG or atomic absorption (Pt). (a) 5TG; (b) Pt. ▲——▲, urine; △——△, serum.

conjunctiva became oedematous and hyperemic with occasional focal haemorrhage. The cornea became slightly oedematous and there was moderate flare and cells in the anterior chambers. *In vitro*, there is measurable slight, toxicity (oxic or hypoxic CHO cells) at 0.5 to 1 $\mu$M. The interaction with radiation is not measureable at this low level using the standard cloning assay.

*Effect of drugs on Greene melanoma*

In control animals, the tumour grew rapidly: nodules appeared on the iris at ~day 5, neovascularization and a thickened iris at ~day 10, with perforation at the limbus at ~day 17. The tumour filled the anterior chamber and large areas of necrosis were observed.

Tumours in the eyes of rabbits treated locally with 5TG commencing at the time of implantation were approximately half the size of controls (Figure 3); those whose treatment began after one week did not show significant growth delay. Some authors have seen an effect of 5TG on tumours (Markoe *et al.*, 1980*b*; Song *et al.*, 1980), yet others found no effect (Rockwell & Schultz, 1984; Tannock *et al.*, 1983). 5TG effectiveness may depend on the site, the detailed metabolic pathways of the tumour cell, the concentration attained and

**Figure 3** Effect of drugs on growth of ocular melanoma. 300 mg of 5TG (twice weekly) or 400 $\mu$g of *cis*-platin (c-Pt) (twice weekly) commencing at time of implantation (group 1) or at 1 week (group 2). Controls received saline only.

the degree of hypoxia. A measurable direct effect of 5TG in ocular melanoma is suggested by our experiments: this needs further characterization.

*Cis*-platin showed a greater effect on tumour growth than 5TG, with an average tumour weight of 0.15 g when drug was started at time of implantation (0.8 g for 5TG) or 0.94 g when treatment was initiated one week after tumour implantation (1.7 g for 5TG) compared with 2.0 g for control animals. This response encourages further investigation of platinum for this system. Experiments using second generation platinum drugs undergoing clinical trials are planned; combinations with other drugs which inhibit tumour growth, e.g. 5-fluorouracil (Rootman *et al.*, submitted) will be assessed; and tumour levels will be measured.

Ocular tumours, while presenting specific difficult challenges to the clinician, offer certain advantages to the researcher. Their growth can be monitored visually and assessed during treatment, at least in a qualitative manner. Local injection of various types of drug may prove to be the route of administration of choice for ocular disease. As well as minimizing systemic toxicity, local administration might have additional advantages over systemic administration for a disease such as retinoblastoma, where second primary neoplasms are frequent which may be related to treatment by drugs or radiotherapy (Draper *et al.*, 1986). In addition, this route of administration offers the opportunity to irradiate when radiosensitizer concentration is maximum (Figure 1). Retinoblastoma contains hypoxic cells (Gallie *et al.*, 1982) which are believed responsible, at least in part, for the failure of local control. Melanoma is reputed to be radioresistant, although this may need reassessment (Harwood & Cummings, 1981). Further evaluation of ocular melanoma for radiobiological studies is warranted and further studies of the interactions between drugs and radiation is planned using retinoblastoma and uveal melanoma cells *in vitro*.

Supported by the B.C. Health Care Research Foundation, Medical Research Council of Canada and the B.C. Cancer Foundation. M.O. Fellenz held a Terry Fox Clerkship (NCI of Canada), summers 1983–84. *Cis*-platin was a generous gift from Dr. P. Simpson, David Bull Laboratories, Victoria, Australia. We are grateful to S.J. Horsky, Geological Sciences, University of B.C., for the atomic absorption analysis.

### References

BUSHWAY, A.A. & WHISTLER, R.L. (1975). Repression of cancer cell growth by 5-thio-D-glucose. *J. Carbo.-Nucleo.*, **2**, 399.

COUGHLIN, C.T., GRACE, M., O'DONNELL, J.F. & 4 others (1984). Combined modality approach in the management of locally advanced head and neck cancer. *Cancer Treat. Reports*, **68**, 591.

DOUPLE, E.B. & RICHMOND, R.C. (1979). Radiosensitization of hypoxic tumour cells by *cis-* and *trans*-dichlorodiammine platinum (II). *Int. J. Radiat. Oncol. Biol. Phys.*, **5**, 1369.

DRAPER, G.J., SANDERS, B.M. & KINGSTON, J.E. (1986). Second primary neoplasms in patients with retinoblastoma. *Br. J. Cancer*, **53**, 661.

GALLIE, B.L., CHEW, E.Y., CHANG, M. & PHILLIPS, R.A. (1982). Retinoblastoma in the eyes of nude mice: Quantitative assessment of therapy. In *Proc. Third Int. Workshop on Nude Mice*, p. 641. New York.

GREENE, H.S. & HARVEY, E.K. (1966). The growth and metastasis of amelanotic melanomas in the heterologous host. *Cancer Res.*, **26**, 706.

HARWOOD, A.R. & CUMMINGS, B.J. (1981). Radiotherapy for malignant melanoma: A reappraisal. *Cancer Treat. Rev.*, **8**, 271.

MARKOE, A.M., BRADY, L.W., EMRICH, J. & 2 others (1980*a*). 5-thio-D-glucose (5TDG) as a radiation sensitizer in hamster pancreatic carcinoma and mouse lung carcinoma models. In *Radiation Sensitizers – Their use in the Clinical Management of Cancer*, Brady, L.W. (ed) p. 282. Masson Publishing Co: New York.

MARKOE, A.M., RISCH, V.R., EMRICH, J. & 2 others (1980*b*). Tissue distribution and retention of 5-thio-D-glucose in animal tumour models. *Ibid*, p. 287.

MOORE, B.A., PALCIC, B. & SKARSGARD, L.D. (1976). Radiosensitizing and toxic effects of the 2-nitroimidazole Ro-07-0582 in hypoxic mammalian cells. *Radiat. Res.*, **67**, 459.

NAGLE, W.A., MOSS, A.J., ROBERTS, H.G. & BAKER, M.L. (1980). Effects of 5-thio-D-glucose on cellular adenosine triphosphate levels and deoxyribonucleic acid rejoining in hypoxic and aerobic Chinese hamster cells. *Radiology*, **137**, 203.

NIAS, A.H.W. (1985). Radiation and platinum drug interaction – Review. *Int. J. Radiat. Biol.*, **48**, 297.

ROCKWELL, S. & SCHULTZ, R.J. (1984). Failure of 5-thio-D-glucose to alter cell survival in irradiated or unirradiated EMT6 tumours. *Radiat. Res.*, **100**, 527.

ROOTMAN, J., BUSSANICH, N. & GUDAUSKAS, G. (1983*a*). Combined local chemotherapy for a spontaneously occurring intraocular tumour in a cat. *Can. J. Ophthalmol.*, **18**, 185.

ROOTMAN, J. & GUDAUSKAS, G. (1985). Treatment of ocular leukaemia with local chemotherapy. *Cancer Treat. Rep.*, **69**, 119.

ROOTMAN, J., GUDAUSKAS, G. & KUMI, C. (1983*b*). Subconjunctival versus intravenous cytosine arabinoside: Effect of route of administration and ocular toxicity. *Invest. Ophthalmol. Vis. Sci.*, **24**, 1607.

ROOTMAN, J., JOSEPHY, P., ADOMAT, H. & PALCIC, B. (1982). Ocular absorption and toxicity of a radiosensitizer and its effect on hypoxic cells. *Arch. Ophthalmol.*, **100**, 468.

ROOTMAN, J., OSTRY, A. & GUDAUSKAS, G. (1984). Pharmacokinetics and metabolism of 5-fluorouracil following subconjunctival versus intravenous administration. *Can. J. Ophthalmol.*, **19**, 187.

SCHULTZ, R.J. & BONGIORNI, P. (1984). On the specific toxicity of 5-thio-D-glucose to hypoxic cells. *Radiat. Res.*, **97**, 352.

SCHUMAN, V.L., LEVITT, S.H. & SONG, C.W. (1982). The radioprotective effect of 5-thio-D-glucose on normal tissues *in vivo*. *Int. J. Radiat. Oncol. Biol. Phys.*, **8**, 589.

SKOV, K.A., FELLENZ, M.O., KUMI, C. & ROOTMAN, J. (1984). The potential of 5-thio-D-glucose for treatment of retinoblastoma (abstr.). *Int. J. Radiat. Oncol. Biol. Phys.*, **10**, 1801.

SONG, C.W., CLEMENT, J.J. & LEVITT, S.H. (1977). Cytotoxic and radiosensitizing effects of 5-thio-D-glucose on hypoxic cells. *Radiology*, **123**, 201.

SONG, C.W., KANG, M.S., STETTNER, S.L. & LEVITT, S.H. (1980). *In vivo* effect of 5-thio-D-glucose on tumour. In *Radiation Sensitizers – Their Use in the Clinical Management of Cancer*, Brady, L.W. (ed) p. 296. Masson Publishing Co: New York.

SONG, C.W., SUNG, J.H., CLEMENT, J.J. & LEVITT, S.H. (1978). Cytotoxic effect of 5-thio-D-glucose on chronically hypoxic cells in multicell spheroids. *Br. J. Cancer*, Suppl. III, **371**, 136.

TANNOCK, I.F., GUTTMAN, P. & RAUTH, A.M. (1983). Failure of 2-deoxy-D-glucose and 5-thio-D-glucose to kill hypoxic cells of two murine tumours. *Cancer Res.*, **43**, 980.